# *ALDH2* polymorphism and alcohol-related cancers in Asians: a public health perspective

**DOI:** 10.1186/s12929-017-0327-y

**Published:** 2017-03-03

**Authors:** Jeffrey S. Chang, Jenn-Ren Hsiao, Che-Hong Chen

**Affiliations:** 1 National Institute of Cancer Research, National Health Research Institutes, 1F. No 367, Sheng-Li Road, Tainan, 70456 Taiwan; 2 Department of Otolaryngology, National Cheng Kung University Hospital, College of Medicine, National Cheng Kung University, 138 Sheng Li Road, Tainan, 70456 Taiwan; 3 Department of Chemical and Systems Biology, Stanford University, School of Medicine, 269 Campus Drive, CCSR Building, Rm. 3140, Stanford, CA 94305 USA

**Keywords:** Aldehyde dehydrogenase 2, Alcohol, Cancer, Prevention, Public health

## Abstract

The occurrence of more than 200 diseases, including cancer, can be attributed to alcohol drinking. The global cancer deaths attributed to alcohol-consumption rose from 243,000 in 1990 to 337,400 in 2010. In 2010, cancer deaths due to alcohol consumption accounted for 4.2% of all cancer deaths. Strong epidemiological evidence has established the causal role of alcohol in the development of various cancers, including esophageal cancer, head and neck cancer, liver cancer, breast cancer, and colorectal cancer. The evidence for the association between alcohol and other cancers is inconclusive. Because of the high prevalence of ALDH2*2 allele among East Asian populations, East Asians may be more susceptible to the carcinogenic effect of alcohol, with most evidence coming from studies of esophageal cancer and head and neck cancer, while data for other cancers are more limited. The high prevalence of ALDH2*2 allele in East Asian populations may have important public health implications and may be utilized to reduce the occurrence of alcohol-related cancers among East Asians, including: 1) Identification of individuals at high risk of developing alcohol-related cancers by screening for *ALDH2* polymorphism; 2) Incorporation of *ALDH2* polymorphism screening into behavioral intervention program for promoting alcohol abstinence or reducing alcohol consumption; 3) Using *ALDH2* polymorphism as a prognostic indicator for alcohol-related cancers; 4) Targeting ALDH2 for chemoprevention; and 5) Setting guidelines for alcohol consumption among ALDH2 deficient individuals. Future studies should evaluate whether these strategies are effective for preventing the occurrence of alcohol-related cancers.

## Background

### Alcohol consumption in the world

According to the 2010 estimate published by the World Health Organization, the global average alcohol consumption for individuals aged 15 or older was 13.5 g of pure alcohol per day [1], which is approximately equivalent to 1 can of beer (12 oz. or 355 ml with 5.0% alcohol content), 1 glass of wine (5 oz. or 148 ml with 12% alcohol content), or 1 drink of distilled spirits (1.5 oz. or 44 ml with 40% alcohol content). The level of alcohol consumption varied by regions with the highest consumption found in Eastern Europe and Russia, and the lowest consumption seen in Southeast Asia, Middle East, and North Africa [1]. Alcohol was consumed most commonly in the form of spirits (50.1%), followed by beer (34.8%) and wine (8.0%) [1]. The WHO projected that by 2025, alcohol consumption will continue to rise, particularly in the Western Pacific Region, which includes countries in the East Asia, unless public health policies are implemented to curb or reverse the trend [1].

### Alcohol-related disease burden

The occurrence of more than 200 diseases can be fully or partially attributed to alcohol drinking [2, 3]. Alcohol use was ranked as the fifth leading risk factor of disease globally in 2010, moving up from eighth place in 1990 [4]. Alcohol-related cancer, liver cirrhosis, and injury accounted for the majority of mortality attributed to alcohol consumption [3]. Together these three conditions caused 1,500,000 deaths worldwide, representing 2.8% of all deaths [3]. The global cancer deaths attributed to alcohol-consumption rose from 243,000 in 1990 to 337,400 in 2010 [3]. In 2010, cancer deaths due to alcohol consumption accounted for 4.2% of all cancer deaths [3]. All evidence indicates that the health impact of alcohol has been on the rise and this trend will likely to continue. In addition, the economic burden associated with alcohol consumption can be tremendous. The annual alcohol-related financial cost was estimated to be US$249 billion for USA, CA$14.5 billion for Canada, GB£21 billion for UK, AU$35 billion for Australia, NT$25.5 billion for Taiwan, and JP¥6300 billion for Japan [5–7].

### Alcohol-related cancers: evidence from epidemiologic studies

Table 1 summarizes the association between alcohol and alcohol-related cancers

**Table 1:** The association between alcohol and alcohol-related cancers

Cancer type	Level of association with alcohol	Summary of current literature
Head and neck cancer	Strong	With light drinking, the risk of head and neck cancer increases by 13%, while the risk increases by more than 5 times with heavy drinking [8].
Esophageal cancer	Strong	With light drinking, the risk of esophageal cancer increases by 26%, while the risk increases by 5 times with heavy drinking [8].
Liver cancer	Strong	Compared to never drinkers, the risk of liver cancer increases by 1.3 times among ever drinkers. With light drinking, the risk of liver cancer increases by 8%, while the risk increases by more than 5 times with heavy drinking [11].
Breast cancer	Moderate	Overall, studies agreed that high level of alcohol consumption is associated with an increased breast cancer risk while the risk of breast cancer at low level of alcohol consumption may require further investigation [8, 12, 13]
Colorectal cancer	Moderate	Light drinking is not associated with colorectal cancer while heavy drinking is associated with a 1.5 times increase in the risk of colorectal cancer [8, 12]

#### Head and neck cancer

The association between alcohol and head and neck cancer is one of the most studied and the results consistently showed an increased head and neck cancer risk associated with alcohol drinking. In a meta-analysis of 52 studies, Bagnardi et al. reported a positive trend in the association between alcohol drinking and head and neck cancer risk (light drinking: relative risk (RR) = 1.13, 95% confidence interval (CI): 1.00–1.26; moderate drinking: RR = 1.83, 95% CI: 1.62–2.07; heavy drinking: 5.13, 95% CI: 4.31–6.10) [8]. In Western countries, approximately 39% of head and neck cancer can be attributed to alcohol consumption (4% for alcohol drinking alone and 35% for the combined effect of alcohol and tobacco) [9].

#### Esophageal cancer

Alcohol consumption is a well-established risk factor of esophageal cancer, particularly esophageal squamous cell carcinoma. In the meta-analysis by Bagnardi et al, alcohol consumption showed a positive dose-response relationship with esophageal squamous cell carcinoma (light drinking: RR = 1.26, 95% CI: 1.06–1.50; moderate drinking: RR = 2.23, 95% CI: 1.87–2.65; heavy drinking: RR = 4.95, 95% CI: 3.86–6.34) [8]. In a meta-analysis of 17 studies by Jarl et al. the risk of esophageal cancer was reduced to the level of never drinkers after 16.5 years of alcohol cessation, suggesting that alcohol cessation is effective in reducing the risk of esophageal cancer [10].

#### Liver cancer

Alcohol is a known risk factor of liver cancer. Chuang et al conducted a meta-analysis of 112 studies and showed an increased risk of liver cancer among ever drinkers compared to never drinkers (RR = 1.29, 95% CI: 1.16–1.42) [11]. They also reported a dose-response relationship for the positive association between alcohol consumption and liver cancer (12 g of alcohol per day: RR = 1.08, 95% CI: 1.04–1.11; 50 g of alcohol per day: RR = 1.54, 95% CI: 1.36–1.74; 75 g of alcohol per day: RR = 2.14, 95% CI: 1.74–2.62; 100 g of alcohol per day: RR = 3.21, 95% CI: 2.34–4.40; and 125 g of alcohol per day: RR = 5.20, 95% CI: 3.25–8.29) [11]. In addition, a synergistic interaction between alcohol drinking and hepatitis or diabetes on the risk of liver cancer was observed [11].

#### Breast cancer

Several meta-analyses have examined the association between alcohol consumption and breast cancer. Jayasekara et al. reported a weak non-linear positive association between alcohol drinking and breast cancer while Bagnardi et al. observed that the risk of breast cancer rose linearly with increasing level of alcohol consumption [8, 12]. Chen et al. reported that 5 g of ethanol from wine per day was associated with a reduced breast cancer risk while the risk of breast cancer began to increase with more than 10 g of ethanol from wine per day [13]. Overall, studies agreed that high level of alcohol consumption is associated with an increased breast cancer risk while the risk of breast cancer at low level of alcohol consumption may require further investigation.

#### Colorectal cancer

Studies have consistently found a positive association between alcohol consumption and colorectal cancer, although the increased risk is modest in magnitude [8, 12]. A meta-analysis by Bagnardi et al. reported a linear positive dose-response relationship between alcohol drinking and colorectal cancer (light drinking: RR = 0.99, 95% CI: 0.95–1.04; moderate drinking: RR = 1.17, 95% CI: 1.11–1.24; heavy drinking: 1.44, 95% CI: 1.25–1.65) [8]. Jayasekara et al. also reported a linear positive dose-response relationship between alcohol drinking and colorectal cancer and the highest level of alcohol consumption was associated with a 1.5 times increase in colorectal cancer risk compared to the lowest level of alcohol consumption [12].

The evidence presented so far only considers the average risk of cancer associated with alcohol consumption. Genetic background may determine an individual’s susceptibility to the carcinogenic effect of alcohol. Individuals deficient in aldehyde dehydrogenase 2 (ALDH2), an enzyme that converts acetaldehyde, a carcinogenic metabolite of ethanol, to acetate, may have a higher risk of alcohol-related cancers. Based on overwhelming epidemiological evidences, the International Agency for Research on Cancer (WHO/IARC) has classified ethanol in alcoholic beverage as a Group 1 chemical, which is carcinogenic to humans [14, 15]. The IARC working group further emphasized the strong evidence of acetaldehyde derived from alcohol metabolism as the mechanistic basis in causing upper aerodigestive track (UADT) cancers in individuals with ALDH2 deficiency [14, 15].

### The role of ALDH2 and its polymorphism

In human, alcohol metabolism involves two major NAD-dependent enzymes, alcohol dehydrogenase (ADH) and aldehyde dehydrogenase (ALDH). Alcohol is first oxidized to acetaldehyde by ADH. Acetaldehyde is then oxidized to non-toxic acetate by ALDH for excretion. Among the different human ALDH isozymes, ALDH2, a mitochondria enzyme, is the most efficient enzyme to remove toxic acetaldehyde [16]. Greater than 90% of the consumed alcohol is detoxified by the first-pass metabolism, especially in the liver where ALDH2 is abundant, although ALDH2 is distributed and functional in many other major organs and tissues [17]. A striking genetic polymorphism that dramatically reduces ALDH2 enzyme activity and affects alcohol response is the variant ALDH2*2 allele. The variant ALDH2*2 allele is caused by a single point mutation (G to A) in exon 12, which leads to an amino acid substitution from glutamine to lysine (E487K) [18]. The normal ALDH2*1 allele and the variant ALDH2*2 allele can be easily genotyped by the determination of single nucleotide polymorphism (SNP) rs671 from the human genome. Due to the tetrameric nature of the ALDH2 enzyme, the E487K mutation exhibits a dominant negative phenotype and affects both heterozygous and homozygous individuals who carry the variant allele. In carriers of the ALDH2*2/*2 homozygous and ALDH2*1/*2 heterozygous genotypes, the enzyme activity is nearly 0% and 17–38% of the normal activity, respectively [19]. Such dramatic reduction in the capacity of acetaldehyde clearance leads to accumulation of acetaldehyde in circulation even after a moderate amount of alcohol intake [20]. Hence, the ALDH2*2 variant causes the well-known Asian Alcohol Flushing Syndrome which is characterized by facial flushing, palpitation, tachycardia, nausea, and unpleasant feelings when alcohol is consumed by these individuals [21]. The ALDH2*2 variant is essentially absent among the Europeans, but is highly prevalent among East Asians [22]. Large scale genotyping and haplotype analysis of the *ALDH2* gene has traced the origin of the ALDH2*2 allele back to the ancient Pai-Yuei tribe about 2000–3000 years ago in Southeast China [23]. The ALDH2*2 allele likely dispersed from its origin toward East Asia with the highest frequencies in Southeastern coastal regions of China (e.g. Guangdong, Fujian provinces) and countries with historical Han migrations, such as Taiwan, Japan, Korea and Singapore [23, 24]. The prevalence of the ALDH2*2 variant varies from 28% (e.g in Korea) to as high as 45% (e.g. in Taiwan) in populations of these regions [23] and an estimated 560 million East Asians are carriers of ALDH2*2 [25]. The ALDH2 deficiency is therefore one of the most common and genetically uniform enzymopathies in human carried by approximately 8% of the world population.

## *ALDH2* polymorphism and risk of alcohol-related cancers

Many studies have investigated the role of *ALDH2* polymorphism and its interaction with alcohol consumption in the development of various cancers. The strongest and most consistent findings have been observed for head and neck cancer and esophageal cancer, while the evidence for other cancers is more limited (Table 2).

**Table 2:** The association between *ALDH2* polymorphism and alcohol-related cancers

Cancer type	Level of association with *ALDH2* polymorphism	Summary of current literature
Head and neck cancer	Strong	Most studies showed a synergistic interaction between ALDH2*2 allele and alcohol drinking to increase head and neck cancer risk [26–29].
Esophageal cancer	Strong	Overall, literature provides strong evidence to support the synergistic interaction between ALDH2*2 allele and alcohol consumption to increase the risk of esophageal cancer [31].
Liver cancer	Weak	Overall, studies to date have consistently shown that ALDH2*2 does not independently contribute to liver cancer risk. Whether ALDH2*2 can modify the association between alcohol consumption and liver cancer risk requires further investigation [32–41].
Breast cancer	Weak	Results from the only three studies published to date do not support a significant association between ALDH2*2 and breast cancer [42–44].
Colorectal cancer	Weak	The role of ALDH2*2 in the development of colorectal cancer is inconclusive and all of the studies conducted to date have a small sample size that resulted in insufficient statistical power and lack of precision [32, 45–47].

### Head and neck cancer

In a meta-analysis of six case-control studies (945 cases and 2917 controls), Boccia et al. reported a reduced risk of head and cancer among individuals with ALDH2*2/*2 genotype, likely due to the lower consumption of alcohol among these individuals [26]. In addition, ALDH2*1/*2 genotype showed gene-environment interaction with the level of alcohol consumption [26]. Compared to ALDH2*1/*1, ALDH2*1/*2 was associated with an increased risk of head and neck cancer among moderate drinkers (odds ratio (OR) = 1.68, 95% CI: 1.27–2.22) and heavy drinkers (OR = 3.57, 95% CI: 1.41–9.05), but not among never drinkers (OR = 0.97, 95% CI: 0.65–1.46) [26]. Three of the four studies published after the meta-analysis all showed a synergistic interaction between ALDH2*2 allele and alcohol drinking to increase head and neck cancer risk [27–29] and only one study did not observe such interaction [30]. Overall, studies have indicated that *ALDH2* polymorphism can modulate the association between alcohol drinking and head and neck cancer risk.

### Esophageal cancer

Zhao et al. conducted a meta-analysis of 31 case-control studies and found that ALDH2*2/*2 was associated with a reduced risk of esophageal cancer (OR = 0.69, 95% CI: 0.48–0.98) while ALDH2*1/*2 was associated with an increased esophageal cancer risk (OR = 2.34, 95% CI: 1.75–3.13) [31]. Individuals with ALDH2*2/*2 tend to avoid alcohol consumption due to the severe reaction after alcohol drinking and this is likely the explanation for their reduced risk of esophageal cancer. However, Zhao et al. reported that among alcohol drinkers, ALDH2*2/*2 was associated with an increased esophageal cancer risk (OR = 3.87, 95% CI: 1.67–8.96) compared to ALDH2*1/*1 [31]. In addition, the association between ALDH2*1/*2 and esophageal cancer became stronger with higher level of alcohol consumption (never drinker: OR = 1.21, 95% CI: 0.95–1.73; light drinker: OR = 3.79, 95% CI: 3.04–4.72; heavy drinker: OR = 6.50, 95% CI: 5.34–7.92) [31]. Overall, literature provides strong evidence to support the synergistic interaction between ALDH2*2 allele and alcohol consumption to increase the risk of esophageal cancer.

### Liver cancer

Among the 10 studies that examined the association between ALDH2*2 and liver cancer risk, 8 found no significant association [32–39], 1 found an increased risk among individuals with at least 1 copy of the ALDH2*2 allele [40], and 1 found an increased risk for heterozygous individuals only [41]. Among the six studies that examined the interaction between ALDH2*2 and alcohol drinking on liver cancer risk, three found a synergistic interaction between ALDH2*2 allele and alcohol consumption to increase liver cancer risk [36, 37, 40], while three found no such interaction [33, 34, 38]. Overall, studies to date have consistently shown that ALDH2*2 does not independently contribute to liver cancer risk. Whether ALDH2*2 can modify the association between alcohol consumption and liver cancer risk requires further investigation.

### Breast cancer

There is limited evidence regarding the association between *ALDH2* polymorphism and risk of breast cancer. To date, only three studies have examined the role of ALDH2*2 in the development of breast cancer and all found no association between ALDH2*2 and risk of breast cancer [42–44]. In addition, Choi et al. and Kawase et al. examined the association between ALDH2*2 and risk of breast cancer stratified by alcohol consumption status and observed no significant interaction between ALDH2*2 and alcohol consumption on the risk of breast cancer [43, 44]. With only three studies published to date, it may be premature to rule out the role of ALDH2*2 in the occurrence of breast cancer; however, evidence from the small number of published studies to date does not support a significant association between ALDH2*2 and breast cancer.

### Colorectal cancer

Four studies, all from Japan, have investigated the association between *ALDH2* polymorphism and colon cancer risk. Yokoyama et al. reported an increased risk of colon cancer (OR = 3.35, 95% CI: 1.51–7.45) associated with alcoholic carriers of ALDH2*2 allele compared to alcoholics with homozygous wild type [32]. Murata et al. reported that alcohol showed a stronger dose-response relationship with colon cancer among ALDH2*1/*2 individuals than among ALDH2*1/*1 individuals, although the results were not statistically significant; in addition, alcohol consumption was not associated with rectal cancer risk regardless of the *ALDH2* genotype [45]. Matsuo et al. reported that high level of alcohol consumption was associated with an increased rectal cancer risk but not colon cancer risk among individuals with ALDH2*1/*2 [46]. Miyasaka et al. found no association between ALDH2*2 and colon cancer and did not assess the interaction between ALDH2*2 and alcohol drinking on colon cancer risk [47]. Overall, the role of ALDH2*2 in the development of colorectal cancer is inconclusive and all of the studies conducted to date have a small sample size that resulted in insufficient statistical power and lack of precision.

## Public health implications

Alcohol flushing and its associated unpleasant feelings due to acetaldehyde accumulation is a strong deterrent against heavy drinking and alcoholism for ALDH2*2 carriers [48]. Many studies have demonstrated the protective effect against alcohol addiction and abuse by ALDH2 deficiency. For example, in 1982, Harada first showed that among Japanese alcoholics, only 2% of the subjects are ALDH2 deficient [49]. However, influenced by social, cultural, and economic factors in the past few decades, such protection against alcohol dependence and alcohol abuse has gradually lost among the large populations of ALDH2*2 carriers. An alarming rise in the proportion of heavy drinkers who are carriers of ALDH2*1/*2 genotype has been documented from the 1970 to 2010. In Japan, the percent of ALDH2*2 alcoholics was 2.5% in 1979, and has increased to 8.0% in 1986, 13.0% in 1992 [50], 13.0% in 1996–2000, 14.0% in 2001–2005, and 15.4% in 2006–2010 [51]. This rapid rise is further highlighted by a recent study in Tokyo area, showing that 26% of the heavy drinking men who consume >400 g of ethanol per week are ALDH2*1/*2 heterozygotes [52]. In Taiwan, an estimation from a 1999 study indicated that 17% of the alcoholics are ALDH2*2 carriers [53]. It is therefore anticipated that, without intervention, the health risk and healthcare burden caused by heavy ALDH2*2 alcohol drinkers will become much more severe in the next few decades. The rapid and dangerous rise in alcohol consumption and dependence among ALDH2*2 carriers also underlines an urgent need for new public health policies and guidelines and active campaigns for public education and awareness in high ALDH2*2 prevalent countries. Given the strong association between *ALDH2* polymorphism and certain alcohol-related cancers, screening for ALDH2*2 allele may have several public health implications, including: 1) Identification of individuals at high risk of developing alcohol-related cancers; 2) Incorporation of *ALDH2* polymorphism screening into alcohol cessation program for promoting alcohol abstinence or reducing alcohol consumption; 3) Using *ALDH2* polymorphism as a prognostic indicator for alcohol-related cancers; 4) Targeting ALDH2 for chemoprevention; and 5) Setting guidelines for alcohol consumption among ALDH2 deficient individuals.

### Identification of individuals at high risk of developing alcohol-related cancers

Early detection is the key to reduce cancer mortality and increase the chance for cure. However, it is not cost-effective to screen the entire population. Because cancer is a rare disease, even if a screening tool has a high sensitivity and specificity, the positive predictive value (PPV) (percent of individuals who test positive and actually have the disease) will still be low. For example, in 2012, East Asia was the region with the highest incidence of esophageal cancer with an incidence of 11 per 100,000 [54]. With this incidence, using a screening tool with 99% sensitivity and 99% specificity will generate a PPV of only 1.1%. This means that for every 100 positive cases detected by screening, only 1 case will actually have esophageal cancer. This is not cost-effective and valuable medical resources will be wasted. In addition, many individuals will undergo unnecessary medical procedures due to positive screening results. To make the screening more cost-effective, it would be important to identify the high-risk population to increase PPV. For example, if we can identify a population with an esophageal cancer incidence of 1 per 100, using a screening tool with 99% sensitivity and 99% specificity, the PPV will increase from 1.1 to 50% (1 of the 2 individuals with a positive screening result will actually have esophageal cancer), which is a substantial improvement in cost-effectiveness. Given the strong evidence for the synergistic interaction between ALDH2*2 allele and alcohol drinking in increasing the risk of several alcohol-related cancers, screening for the carriers of ALDH2*2 allele may be ideal for identifying individuals at high risk for alcohol-related cancers. For example, Yokoyama et al. have developed a health risk model for screening esophageal cancer [55, 56]. The model included information on alcohol drinking, *ALDH2* genotype or facial flushing after alcohol consumption, which is a physical symptom strongly associated with ALDH2*2 allele, cigarette smoking, and consumption of vegetables and fruits [55, 56]. Using this health risk model, Yokoyama et al. were able to identify individuals with higher risk of developing esophageal cancer, with an esophageal cancer detection of 2.9% in the high-risk group compared to 0.5% in the low-risk group [56]. Another study by Koyanagi et al. built a risk prediction model of UADT cancer that incorporated information on sex, age, alcohol drinking, cigarette smoking, and *ALDH2* genotype and reported that the risk model had a good discriminative ability with an area under the curve of more than 0.8 [57]. In addition, they reported that heavy drinkers with ALDH2*1/*2 genotype have a 20% cumulative risk of developing UADT cancer by the age of 80 years, while other individuals have < 5% risk of developing UADT cancer by the age of 80 years [57]. These studies suggest that *ALDH2* genotype may be incorporated in a risk prediction model to identify individuals at high risk of developing alcohol-related cancers, particularly those cancers that are strongly influenced by gene-environment interaction between alcohol and *ALDH2*, such as head and neck cancer and esophageal cancer. For other alcohol-related cancers, including liver cancer, breast cancer, and colorectal cancer, for which the evidence for the involvement of *ALDH2* is more limited, more studies are needed to evaluate the interaction between *ALDH2* polymorphism and alcohol drinking on the risk of these alcohol-related cancers.

### Incorporation of *ALDH2* polymorphism screening into alcohol cessation program

It is possible that the knowledge regarding *ALDH2* genotype and the associated disease risk may motivate individuals to reduce alcohol consumption. Hendershot et al. conducted a web-based genetic feedback intervention trial incorporating information on *ALDH2* genotype [58]. They recruited 200 northeast Asian American college students and randomized them to control and intervention groups. Individuals in the control group received a web-based feedback session that included normative information about behaviors of college students [58]. Individuals in the intervention group received different web-based feedback sessions according to their *ALDH2* genotype. Individuals with ALDH2*1/*1 received risk information on alcohol dependence while individuals with ALDH2*1/*2 received risk information on alcohol-related cancers [58]. One month after the web-based feedback session, individuals in the intervention group showed significant reduction in the frequency and the quantity of alcohol drinking while individuals in the control group showed no significant change in drinking behaviors [58]. This suggested that it may be feasible to incorporate information on *ALDH2* genotype and the associated disease risk into an alcohol cessation program to effectively reduce the consumption of alcohol. However, the use of genetic information to change health behaviors is complex and its effectiveness is still inconclusive [59]. For example, Smerecnik et al. conducted a meta-analysis of nine studies and found that genetic testing of genes associated with smoking-related diseases had only a short-term effect on risk perception and motivation to quit smoking, which did not last with longer follow-ups [60]. More studies are needed to determine whether genetic testing of *ALDH2* will achieve a long lasting effect for reducing the consumption of alcohol. It is possible that the outward physical symptoms, including facial flushing, palpitation, tachycardia, and nausea, associated with ALDH2*2 may serve as a strong reminder to maintain the motivation for reducing alcohol consumption. In addition, more investigations are needed to determine the optimal and culturally appropriate methods for delivering genetic information and the related disease risks.

### *ALDH2* polymorphism as a prognostic indicator for alcohol-related cancers

*ALDH2* polymorphism may be used to predict the survival, recurrence, and development of second-primary or other alcohol-related cancers for patients with alcohol-related cancers, particularly for esophageal cancer and head and neck cancer. Kawakita et al. reported that both alcohol drinking and ALDH2*2 were not independently associated with disease-free survival of head and neck cancer [61]. However, there was a significant positive dose-response between higher alcohol consumption and poorer disease-free survival of head and neck cancer among ALDH2*1/*1 individuals [61]. The exact biological mechanism underlying this relationship was not clear and the authors speculated that patients with ALDH2*1/*1 might continue to drink alcohol at a higher level after head and neck cancer treatment, resulting in their poorer outcomes [61]. Yokoyama et al. conducted a follow-up study of 100 alcoholic men diagnosed with esophageal squamous cell carcinoma and observed that during the follow-period, individuals with ALDH2*1/*2 were at higher risk of developing metachronous squamous cell carcinoma in the esophagus, oral cavity, pharynx and larynx (age-adjusted hazard ratio = 3.38, 95% CI: 1.45–7.85; alcohol-adjusted hazard ratio = 4.27, 95% CI: 1.42–12.89) compared to individuals with ALDH2*1/*1 [62]. Due to limited number of studies investigating the role of ALDH2*2 in the survival or recurrence of alcohol-related cancers, it may be premature to consider its clinical application. However, once such role of ALDH2*2 has been proven, the follow-up schedule and methods for alcohol-related cancer patients may be tailored according to their *ALDH2* genotypes to increase survival and improve early detection of cancer recurrence or second primary cancer.

### Setting guidelines for alcohol consumption among ALDH2 deficient individuals

If an individual does not drink alcohol, the best recommendation for alcohol consumption would be not to start. If one does drink, the Dietary Guidelines for Americans 2015–2020 recommends no more than 1 drink (14 g of pure alcohol) per day for women and 2 drinks for men [63]. The definition of low-risk alcohol consumption varies widely across different countries in the world ranging from 10 g per day to 42 g per day for women and 14 g per day to 56 g per day for men [64]. These guidelines do not consider the influence of *ALDH2* polymorphism on the association between alcohol drinking and cancer and the amount of alcohol that is considered low risk may not be suitable for individuals with ALDH2 deficiency. For example, in a study by Lee et al., compared to never drinkers with ALDH2*1/*1, drinking < 30 g of alcohol per day was associated with a 2.2 times increase in esophageal cancer risk for those with ALDH2*1/*1, but the risk increased by 14.5 times and 17.3 times, respectively, for those with ALDH2*1/*2 and ALDH2*2/*2 [65]. This showed that even with the amount of alcohol considered as “light” or “moderate” drinking, the increase in esophageal cancer risk could be substantial for carriers of ALDH2*2 allele. Therefore, when setting the level of low-risk alcohol consumption, it is important to consider the increased susceptibility to the carcinogenic effect of alcohol among carriers of ALDH2*2 allele. This is particularly relevant for East Asian countries where the prevalence of ALDH2*2 allele is high. More studies are needed to quantify the dose-response relationship between alcohol consumption and risk of various cancers by *ALDH2* genotypes, particularly for esophageal cancer and head and neck cancer, for both the risk is significantly increased by the synergistic interaction between alcohol consumption and ALDH2*2 allele. Results from these studies will be important for experts in the field and public health officials to decide on the level of alcohol consumption that confers minimum acceptable risk.

### Chemoprevention by aldehyde dehydrogenase activators

Exposure to acetaldehyde, a volatile, reactive and commonly present compound from either physiological conversion of alcoholic beverages or from the environmental sources (such as cigarette smoke [66], food items [67], automobile exhausts [68] etc.) is almost unavoidable. Considering the carcinogenicity of acetaldehyde and the significantly increased risk for head and neck cancer, esophageal cancer, and other cancers for ALDH2*2 variant carriers, implementation of chemopreventive strategies may be a worthwhile effort for specific high-risk groups, such as ALDH2*2 individuals who are heavy drinkers or smokers or industrial workers who have a higher acetaldehyde exposure burden. One of the chemopreventive strategies is enhancing the catalytic activity of the aldehyde dehydrogenase enzyme for faster acetaldehyde clearance. Recently, a group of novel small molecule ALDH enzyme activators have been discovered. These compounds may serve as pharmaceuticals leads for the development of chemopreventive agents based on their enhanced capability of acetaldehyde removal. Alda-1 (N-(1,3- benzodioxol-5-ylmethyl)-2,6-dichlorobenzamide) was the first identified potent selective activator for ALDH2 [69]. The compound has been tested and shown to be effective in several animal models of human diseases related to excessive aldehyde toxicity [70–72]. One of the unique features of Alda-1 is that the compound not only increases the catalytic activity of the wild type ALDH2*1 enzyme, but can also correct the defect of the mutant ALDH2*2 enzyme [69, 73]. This feature is particularly useful for the development of a chemopreventive drug specifically for the ALDH2*2 carriers and the targeted high-risk human groups [74]. Another useful ALDH activator is Alda-89 (5-(2-propenyl)-1,3-benzodioxole) which was discovered as a selective ALDH3A1 isozyme activator [75]. The ALDH3A1 is not an enzyme that metabolizes acetaldehyde efficiently under normal physiological condition. However, with the addition of Alda-89, the catalytic activity of ALDH3A1 toward acetaldehyde could be increased by at least 5 folds [75, 76]. In an acute alcohol intoxication animal model, it was demonstrated that Alda-89 was able to recruit ALDH3A1 to assist the function of ALDH2 for acetaldehyde detoxification in both wild type and ALDH2*2 knock-in mice [76]. It is conceivable that ALDH activators, like Alda-1 or Alda-89, can be developed in various forms for rapid clearance of acetaldehyde regardless of its source or presence in either saliva or circulating blood. Such drugs could potentially have a great utility in reducing the risk of cancer or cancer recurrence, especially among the groups of high-risk individuals who are alcohol drinkers, cigarette smokers and carriers of ALDH2*2 allele. Caution should also be given in the design of a chemopreventive program using aldehyde dehydrogenase activators, since it is not clear whether by enhancing ALDH activity, there will also be a risk of increased alcohol consumption among ALDH2*2 subjects. Behavioral intervention studies and risk assessment on alcohol consumption and cessation similar to the study mentioned above by Hendershot et al. [58] should be recommended and incorporated in the design of such a chemopreventive program.

## Conclusions

Given the rising consumption of alcohol around the world, the incidence of alcohol-related cancers will continue to rise, particularly in East Asian countries and regions with high concentrations of East Asian immigrants, where the percentage of ALDH2 deficient individuals is high. Information regarding an individual’s *ALDH2* genotype may help establish effective public health preventive strategies to reduce the occurrence and improve the survival of alcohol-related cancers. Screening for carriers of ALDH2*2 allele may identify high-risk individuals for alcohol-related cancers. These high-risk individuals may be targeted for more frequent screening of alcohol-related cancers, health education, and alcohol cessation program. In addition, alcohol-related cancer patients carrying ALDH2*2 may be at higher risk for recurrence or developing second primary cancer and thus follow-up plan with more clinical visits may be required. Finally, chemo-preventive agent to restore the function of ALDH2 enzyme may be considered to prevent the occurrence or the recurrence of alcohol-related cancers among high-risk individuals.

## Abbreviations

ADH: Alcohol dehydrogenase
ALDH: Aldehyde dehydrogenase
CI: Confidence interval
IARC: International Agency for Research on Cancer
OR: Odds ratio
PPV: Positive predictive value
RR: Relative risk
UADT: Upper aerodigestive tract.
